# Blood oxygenation during hyperpressure intraperitoneal fluid administration in a rabbit model of severe liver injury: Evaluation of a novel concept for control of pre-hospital liver bleeding

**Published:** 2012

**Authors:** Siavash Ahmadi-Noorbakhsh, Saeed Azizi, Bahram Dalir-Naghadeh, Masoud Maham

**Affiliations:** *Department of Clinical Sciences, Faculty of Veterinary Medicine, Urmia University, Urmia, Iran.*

**Keywords:** Intra-abdominal Hypertension, Liver, Hemorrhage, Pre-hospital Care, Rabbit

## Abstract

Oxygen is an essential part of the most important metabolic pathways in aerobic organisms. Oxygen delivery is merely dependent on blood, rendering blood loss a devastating event. Traumatic pre-hospital liver bleeding is a major cause of early trauma deaths in human and animals, with no established therapeutic method yet. Increasing intra-abdominal pressure (IAP) has been shown to reduce liver bleeding by half. Although reduction of blood loss could be in favor of blood oxygen delivery, however, the complex interaction between increased IAP and respiratory mechanics during severe hemorrhagic shock remained unclear. We used a novel model of liver trauma in 16 rabbits and randomly assigned them to either normotensive abdomen group or increased IAP by fluid infusion (HA) groups (n=8 each). Liver size and the amount of liver injury were evaluated. Various blood oxygenation parameters were recorded. Both groups were identical in terms of the liver size and injury. The HA group had significantly lower shock index. Arterial oxygen capacity and oxygen content were higher in the HA group. No significant statistical difference was seen between groups in terms of abdominal perfusion pressure; alveolar pressure of oxygen; dissolved oxygen in blood plasma; alveolar to arterial oxygen tension gradient; arterial to alveolar oxygen pressure ratio; the ratio between partial pressure of arterial oxygen and fraction of inspired oxygen; and respiratory index. In conclusion, the novel therapeutic method of increasing IAP by fluid infusion in a rabbit model of liver hemorrhage preserved blood oxygenation better than the classic therapeutic method.

## Introduction

Oxygen is a key element in aerobic cell metabolism. In a clinical point of view, oxygen content of blood and oxygen delivery are of the most important issues in critically ill and unconscious patients.^1^ Indeed, inadequate oxygen supply leads to hypoxia, which if left undetected or not treated appropriately may lead to high morbidity and mortality. Hypoperfusion hypoxia and hypoxic hypoxia are two main categories in the classification of hypoxia.^2^

In a close analogy, shock is defined as “failure to maintain tissue perfusion and oxygenation”.^3^ Hemorrhagic shock is one of the most common types of shock following trauma, with a high mortality rate, especially in the pre-hospital setting.^3^^,^^4^ Hemorrhagic shock could be a mixture of various kinds of hypoxia, including hypoxic hypoxia (due to diffusion impairment, ventilation/perfusion mismatch, etc.), anemic hypoxia, and hypoperfusion hypoxia.^2^ Thus, determination and restoration of blood oxygenation and oxygen delivery is the main objectives in traumatic hemorrhaging patients.^3^ In a previous study of traumatic liver hemorrhage, we have shown great reduction of liver bleeding following hyperpressure intra-abdominal fluid infusion via a small catheter. In fact, hyperpressure fluid in the abdominal cavity acts like pushing a tampon on the bleeding site.^5^ This method is useful in pre-hospital arena where there is no direct intra-abdominal access. Although increasing the intra-abdominal pressure (IAP) is not a definitive cure, it can buy time until the patient can be brought to the medical center for definitive treatment. While this new concept promises an exciting future for the pre-hospital arena, still arguments remained about the negative effects of intra-abdominal hypertension (IAH) on the respiratory system. The rationale is that increased IAP mechanically displaces the diaphragm towards the thorax. This may lead to alveolar collapse, decreased functional residual capacity, and decreased tidal volume.^6^ Thereby, although IAH reduces the hemorrhage (leading to increased blood oxygenation), however at the same time it may negatively affect the respiration (leading to decreased blood oxygenation). Therefore, the respiratory effect of increased IAP for controlling traumatic intra-abdominal liver bleeding is a complex event and behaves like a two-edged sword: its benefits should be weighed against its detrimental effects. 

The aim of this study was to elucidate effects of fluid induced intra-abdominal hypertension on blood oxygenation in a mixed model of intra-abdominal hemorrhage following severe experimental liver injury in rabbits. Results of this study may have implications on management of veterinary or medical patients with concurrent traumatic intra-abdominal bleeding and pathologic IAH (e.g. bowel edema due to heavy fluid resuscitation), or laparoscopic surgeries of traumatic patients in which a method of abdominal insufflation is routinely used.

## Materials and Methods

This study was approved by the Urmia University Research Council and conformed to the rules of the Protection of Vertebrate Animals Used for Experimental and other Scientific Purposes.^7^ Sixteen male New Zealand White rabbits (2.085 ± 0.185 kg) were used in this study. The animals were randomly divided into two groups: Normotensive abdomen (NA) as controls and hypertensive abdomen (HA) as treatment groups, with 8 animals in each group. Details of surgical procedures and liver injury modeling have been reported previously,^5^ and are briefly described here. Intravenous access was established through marginal ear vein. 

Rabbits were tranquilized by slow intravenous ketamine (15 mg kg^-1^, Alfasan, Holland) injection. Anesthesia was deepened by halothane face mask. Orotracheal intubation was performed, and a modified Jackson-Rees non-rebreathing anesthesia circuit was used for maintenance of anesthesia.^8^ Right carotid artery was catheterized for arterial pressure monitoring and blood sampling. Liver was injured with a novel method under complete control on the IAP.^5^ Briefly, two tourniquets were preplaced around the vascular structures of the left lateral lobe of the liver: (1) proximally: a single loose tourniquet to stop the bleeding at the end of the experiment, and (2) distally: a modified Rumel tourniquet to temporarily delay the bleeding after liver transection while the abdomen was open. Heparin (800 IU kg^-1^; Rotex Medica, Germany) was administered intravenously. The tourniquet was gently tightened. The liver lobe was resected 20% up its proximal length. Abdominal incision was closed and the tourniquet was removed to initiate the hemorrhage. The animals were promptly randomized to either NA group, or HA group.

The animals of NA group only received IV lactated Ringer’s fluid resuscitation (the classic method of pre-hospital care for liver hemorrhage patients) and the animals of HA group received IV lactated Ringer’s fluid resuscitation along with IP infusion of 3.86% dialysate (type III peritoneal dialysis solution) to produce an IAP of 11 mm Hg. The aforementioned IV lactated Ringer’s fluid resuscitation was used to keep the mean arterial blood pressure (MAP) above 30 mmHg according to the reference value (69 ± 4 mL kg^-1^ in NA group and 0 mL kg^-1^ in HA group).^5^ The procedure was continued for 20 min.

Data were recorded at baseline (exactly before constricting the Rumel tourniquet and injuring the liver) and at 5-min intervals after initiation of bleeding.^5^ Abdominal perfusion pressure (APP; mmHg)^9^ and modified shock index (SI)^10^ were recorded by a computerized data acquisition system (PowerLab ML866 4/30; ADInstruments, Spechbach, Germany) according to the reference methods. Alveolar pressure of oxygen (mmHg; PAO_2_), alveolar to arterial oxygen tension gradient (AaDO_2_; mmHg), arterial to alveolar oxygen pressure ratio (aA Ratio; mmHg), oxygen capacity (O_2_Cap; mL dL^-1^), oxygen content (O_2_Ct; mL dL^-1^), the ratio between partial pressure of arterial oxygen (PaO_2_) and fraction of inspired oxygen (PaO_2_/FiO_2_; mmHg), and respiratory index (RI) were obtained from an arterial blood gas analyzer (Stat Profil^®^ pHOx^®^ Plus; Nova Biomedical, USA). Dissolved oxygen in blood plasma (DissO_2_; mL dL^-1^) was calculated by subtracting the O_2_Cap from the O_2_Ct.^3^^,^^11^ Since the liver has a high oxidative metabolic rate,^12^ to ensure that animals were homogenous in terms of liver size and mass, we measured the maximum length of the liver lobe and generated a liver weight (LIW) profile. The LIW profile was defined as the total LIW, total lobe weight, ratio of the lobe weight to the LIW, and ratio of LIW to pre-injury body weight (Table 1). To ensure about the identical amount of the liver injury in both study groups, the weight profile of transected liver parts was evaluated according to the following parameters and ratios: resected lobe weight (RLW), remaining LIW (total LIW minus RLW), ratio of RLW to remaining LIW, ratio of RLW to total LIW, and ratio of RLW to pre-injury body weight (Table 2). At the end of the experiment, blood loss measurement was performed according to the reference method.^5^ Briefly, the hematocrit, red blood cell count, and hemoglobin of the abdominal fluid samples were measured. Knowing the volume of the abdominal fluids, we calculated total RBC volume loss, total RBC count loss, and total Hb loss according to the reference formulas.^5^

Histograms, boxplots, scatter plots, and Shapiro–Wilk test were used to assess the data for outliers, normality of distribution and homogeneity of variances. Data for AaDO_2_; PaO_2_/FiO_2_; DissO_2_; RI; liver lobe maximum length; ratio of the lobe weight to the LIW; ratio of LIW to pre-injury body weight; ratio of RLW to remaining LIW; ratio of RLW to total LIW; and ratio of RLW to pre-injury body weight were not normally distributed which were normalized by natural logarithm. Seven covariance structures (first-order autoregressive, heterogeneous first-order autoregressive, first-order ante-dependence, Toeplitz, unstructured, compound symmetry, and heterogeneous compound symmetry) were examined. The Toeplitz structure was fitted for DissO_2_. Rests of the parameters were evaluated by first-order ante-dependence. 

Two-way repeated measures ANOVA was used to test overall differences between groups and within subjects, followed by Bonferroni test for multiple pairwise comparisons. Differences between groups for the variables related to the liver weight profile and the weight profile of transected liver parts were tested by unpaired Student’s t test. A value of *P* < 0.05 was considered significant. Results are presented as mean ± SEM. Statistical analyses were conducted using PASW Statistics release 18 for Windows (SPSS Inc. Chicago, USA).

## Results

**Fig. 1:**
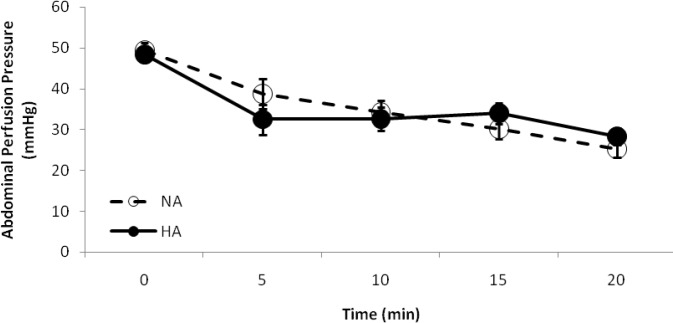
Reduction of abdominal perfusion pressure (APP). Although APP was significantly reduced in both groups (*P *< 0.0001), it followed a constant reduction rate in the NA group while a stepwise fashion in the HA group. NA, normotensive abdomen; HA, Hypertensive abdomen

The blood loss in the HA group was significantly lower than that in the NA group (*P *< 0.0005). No statistically significant difference in PaO_2_ or hemoglobin oxygen saturation levels was observed between two groups. The APP in both groups was significantly reduced between the baseline and final states (*P *< 0.0001). For NA group, the APP was gradually reduced in an approximately constant rate (Fig. 1). For the HA group, just after induction of iatrogenic intra-abdominal hypertension, the APP was significantly reduced from 48.42 ± 1.88 mmHg at baseline, to 32.47 ± 3.70 at 5 min (*P *= 0.009).

**Fig. 2:**
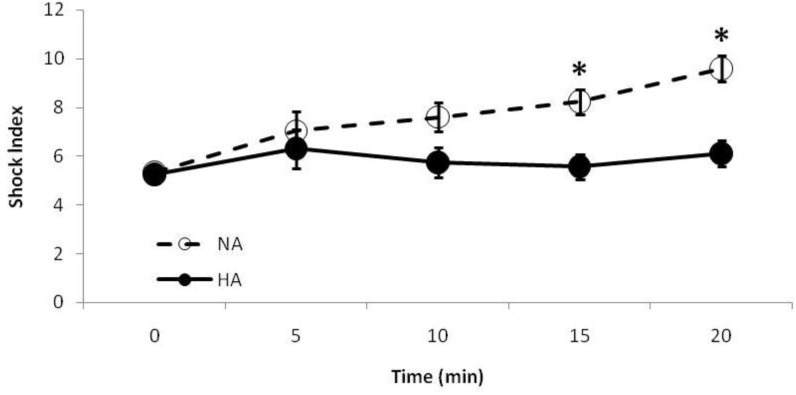
Trend of shock index through the course of the experiment. Shock index in the HA group was relatively constant, but it was significantly increased in the NA group (*P *< 0.0001) implicating on a worse outcome. NA, normotensive abdomen; HA, Hypertensive abdomen

The APP remained in an almost steady state for the remaining part of the experiment. No significant statistical difference of APP was observed between the NA and HA groups. Shock index in the NA group (Fig. 2) was significantly increased through the course of the experiment (5.32 ± 0.28 at baseline versus 9.59 ± 0.53, at 20 min; *P *< 0.0001). The HA group had a relatively constant values of SI through the experiment, without significant differences between the baseline and final states (*P *= 0.88). The HA group had lower shock index values than the NA group, with statistically significant differences at the 15- and 20-min time points (*P *< 0.04). 

**Fig. 3:**
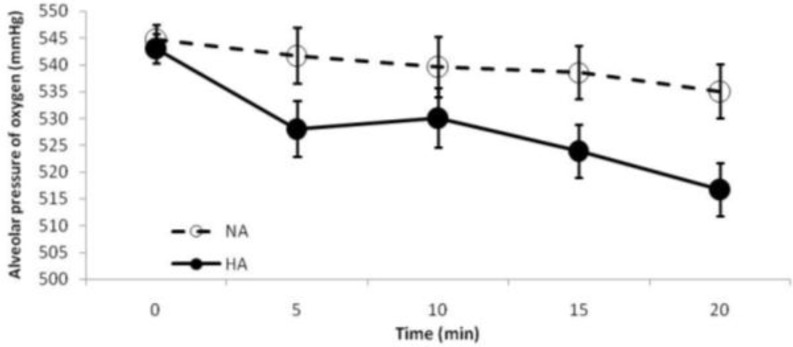
Alveolar pressure of oxygen. It was significantly reduced in the HA group between its baseline and final states (*P *= 0.002). No statistically significant difference was observed between groups. NA, normotensive abdomen; HA, Hypertensive abdomen

The value of PAO_2_ is presented in Figure 3. There was no statistically significant difference between groups, however, final PAO_2_ level of HA group was significantly lower than the baseline value of this group (*P* = 0.002). 

**Fig. 4:**
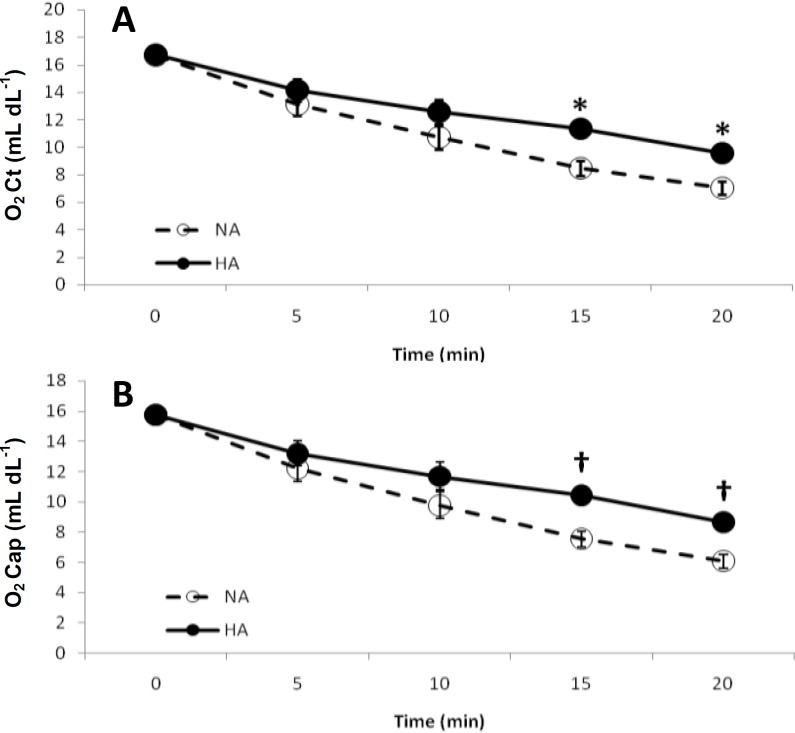
Trends in O_2_Ct (A) and O_2_Cap (B). There were similar trends for both parameters. Both parameters were significantly reduced between the baseline and final states (*P *< 0.0001). NA, normo-tensive abdomen; HA, Hypertensive abdomen. * *P* < 0.04; † *P* < 0.03.

The amounts of O_2_Cap and O_2_Ct were changed in a similar fashion (Fig. 4). There was a significant reduction in O_2_Cap and O_2_Ct in both experimental groups (*P *< 0.0001), however, in minutes 15 and 20, the HA group had significantly higher values of O_2_Cap (*P* < 0.03) and O_2_Ct (*P* < 0.04) than the NA group. The amount of DissO_2_ was statistically similar between groups and through the course of the experiment. No statistically significant change was observed for AaDO_2_, aA Ratio, PaO_2_/FiO_2_, and RI between two study groups or between their baseline and final states (data not shown).

Table 1 shows the liver size and weight profile of the groups. No statistically significant differences were observed between the groups in terms of maximum length of the liver lobe and the LIW profile. All the measured parameters related to the amount of the liver injury were statistically identical between groups (Table 2).

**Table 1:** Liver weight profiles of the NA and HA groups. All parameters are presented as the mean ± SE.

	**Max. Lobe Length (cm)**	**Total LIW (g)**	**Total Lobe Weight (g)**	**Lobe Weight to LIW (%)**	**LIW to Body Weight (%)**
**HA**	7.60 ± 0.20	73.90 ± 3.80	18.10 ± 2.20	24.00 ± 1.50	3.56 ± 0.17
**NA**	7.60± 0.30	63.90 ± 3.40	16.70 ± 1.10	26.00 ± 0.74	3.16 ± 0.18

No statistically significant difference was seen between groups

(*P *> 0.05). LIW: Liver weight, HA: Hypertensive abdomen group, NA: Normotensive abdomen group.

**Table 2:** Transected liver weight profiles of the NA and HA groups. All parameters are presented as the mean ± SE

	**RLW ** **(g)**	**Remaining LIW (g)**	**RLW to Remaining LIW (%)**	**RLW to ** **Total LIW ** **(%)**	**RLW to ** **Body Weight ** **(%)**
**HA**	14.40 ± 1.30	59.50 ± 3.00	26.59 ± 0.88	19.50 ± 1.13	0.70 ± 0.06
**NA**	13.40 ± 1.10	50.50 ± 2.30	26.23 ± 1.02	20.74 ± 0.63	0.66 ± 0.05

No statistically significant difference was seen between groups

(*P *> 0.05). RLW, Resected lobe weight; LIW: Liver weight, HA: Hypertensive abdomen group, NA: Normotensive abdomen group.

## Discussion

Our study showed that the novel method of iatrogenic intra-abdominal hypertension with a certain level of IAP during hemorrhagic shock did not negatively affect the parameters of blood oxygenation in comparison to the group with normal intra-abdominal pressure and hemorrhagic shock. In this study, the SI, APP, O_2_Cap, and O_2_Ct values were in favor of the HA group, while there was no statistically significant difference among the groups in terms of AaDO_2_, aA Ratio, PaO_2_/FiO_2_, RI, and DissO_2_.

Previous researches used an IAP of 20 cm H_2_O (14.7 mmHg) for controlling intra-abdominal hemorrhage in pigs.^14^^-^^16^ In our pilot study, this IAP resulted in a failure of respiratory movements and deleterious blood gas parameters, suggesting the influence of differing respiratory muscle strength between pigs and rabbits. Although the aim of this study was not to determine the ideal IAP for the control of liver bleeding, no severe respiratory effects were seen after the IAP was decreased to 11 mmHg. It should be noted that an IAP of 10 mmHg in rabbits is defined as the routine IAP for a laparoscopic study.^17^

The APP is an important indicator of abdominal organ perfusion and implicate on oxygen delivery, especially during intra-abdominal hypertension.^18^ In critically ill patients, it has been shown that APP has a higher predictive value than other routine resuscitation end-points such as IAP, arterial pH, base excess (or base deficit), and arterial lactate content.^19^ Since it is calculated by subtracting the IAP from MAP^9^, the reduction of APP in both groups is directly related to the reduction of MAP during hemorrhagic shock. The significant reduction of APP in the HA group between the baseline and 5 min time point could be related to rapid increase of IAP at the beginning of the experiment. Afterwards, it seems that compensatory mechanisms attempted to maintain the APP in a relatively constant level, by preventing more MAP reduction. No significant difference of APP among the study groups may implicate on both groups having a similar blood perfusion in abdominal organs. 

The shock index is a predictive value for the severity of blood loss, shock and survivability. It was developed for prompt assessment of hemorrhagic patients^10^ and it is known to be a better predictor for identifying critically ill patients than either heart rate or systolic or diastolic blood pressure alone.^3^ The lower SI in the HA group may show better oxygenation due to less severe blood loss and shock, and an overall less critical condition in this group. Comparing both APP and SI, it may be concluded that although HA and NA groups had a similar abdominal organ perfusion, however, the HA group had an overall better oxygenation than the NA group. This phenomenon may be related to the shifting of blood from the abdominal compartment (and possibly caudal limbs) toward the thorax and cranial body regions. Although this may result in reduction of abdominal perfusion, however, this is in cost of better oxygenation to more vital structures such as brain and heart. The amount of PAO_2_ could be mathematically presented by the alveolar gas equation:^20^

${\mathrm{PA}}_{\mathrm{o2}}=\left(P_{B}-P_{H_{2}o}\right).{\mathrm{Fi}}_{\mathrm{o2}}-\frac{{\mathrm{Pa}}_{\mathrm{co2}}}{R}$

Where, P_B_ is the barometric pressure, which for the Urmia city with 4264 feet above the sea level, P_B_ would be about 650 mmHg; PH_2_O is the pressure of water vapor at 37°C and is about 47 mm Hg; FiO_2_ is the fraction of inspired oxygen (in decimal number); PaCO_2_ is the partial pressure of carbon dioxide; and R is the respiratory exchange ratio, usually considered to be 0.8.^11^ In our study, the only unfixed parameter of this equation was the PaCO_2_ level, with significantly higher value in the final state of the HA group.^5^ Due to high diffusion rate of carbon dioxide through the alveolar membrane, it is assumed that the partial pressure of alveolar carbon dioxide is very close to PaCO_2_.^11^ Thereby, according to Dalton’s law of partial pressures^21^ increased PaCO_2 _in the HA group was resulted in decreased PAO_2_. At first look, it seems to be a miserable event, however, by considering the O_2_Cap^22^ and the O_2_Ct (discussed at length below), it can be seen that the lower PAO_2 _in the HA group was not clinically significant in terms of blood oxygenation and indeed the HA group was significantly more oxygenated than the NA group. The O_2_Ct is an integration of the primary blood parameters involved in oxygen delivery, including arterial hemoglobin oxygen saturation, hemoglobin concentration, and the PaO_2_,^23^^,^^24^ with two former parameters having more influence than the latter. Therefore, considering insignificant change of hemoglobin oxygen saturation and the PaO_2_ in our study groups,^5^ the reduction of the O_2_Ct through the course of the experiment seems to be mainly related to decreased hemoglobin concentration (hemodilution resulted from fluid resuscitation in the groups). Higher values in the HA than the NA group, might have resulted from lower hemorrhage in the HA group. This rationale can be confirmed confirmed by analyzing the O_2_Cap. This parameter is the capacity of blood to carry oxygen in the form of oxyhemoglobin. O_2_Cap was higher in the HA group than the NA group, showing that the HA group had a higher hemoglobin concentration. The higher values of O_2_Ct than the O_2_Cap were mainly due to the DissO_2_^3^^,^^13^ which contributes to the partial pressure of arterial oxygen.^11^

No statistically significant change of AaDO_2_, aA Ratio, and PaO_2_/FiO_2_ among the groups may implicate on no intrapulmonary shunt perfusion after induction of IAH in the HA group.^11^^,^^25^^,^^26^ The RI covers a wide variety of “factors determining the exchange of oxygen between inspired gas and blood” and is calculated as follows:^11^

$\mathrm{RI}=\frac{\left({\mathrm{PA}}_{\mathrm{o2}}-{\mathrm{Pa}}_{\mathrm{o2}}\right)}{{\mathrm{Pa}}_{\mathrm{o2}}}$

Non-significant change of this parameter probably negates malfunctions in oxygen exchange. No statistical difference between groups in terms of liver mass or size, or amount of liver injury might confirm that observations in this study could not be attributed to individual differences of rabbits in terms of liver size, liver mass, liver trauma *per se*, or technique of liver trauma modeling.

This study had some limitations. Due to technical reasons we could not measure cardiac output, although it had been shown that oxygen delivery was better described as a function of both O_2_Ct and the cardiac output.^3^ However, according to normal RI level in the HA group, and since the RI is sensitive to changes in cardiac output,^11^ it could be extrapolated that the cardiac output of the HA group was not significantly different from its normal values. Also, we did not measure mixed venous blood samples due to technical reasons and negative biasing effects that high volume blood samplings could have in our experiment. Since the aim of this experiment was not to evaluate oxygen consumption by tissues, we believe that this limitation did not influence our current findings.

In conclusion, our study on a mixed model of hemorrhage and iatrogenic IAH showed that the novel therapeutic method consisting of iatrogenic IAH, in comparison with classic method of resuscitation consisting of normal IAP, better preserves blood oxygenation parameters. This study may corroborate the safety and effectiveness of the novel concept of therapeutic intra-abdominal hyper-tension for control of severe liver bleeding. Our results may also have implications in patients with traumatic liver bleeding and concurrent pathologic IAH, or laparoscopic surgeries of traumatic liver injury patients in which abdominal insufflation is used routinely.
